# Thrombotic antiphospholipid syndrome in a child with human immunodeficiency virus: a rare case report

**DOI:** 10.1186/s12959-021-00273-y

**Published:** 2021-03-25

**Authors:** Rong-Jing Dong, Su-Yun Lei, Jun Li, Xin-Ping Yang, Yu-Ye Li, Yun-Gui Zhang

**Affiliations:** 1 Yunnan Provincial Hospital of Infectious Disease/Yunnan AIDS Care Center, Kunming, 650301 China; 2 Department of Dermatology and Venereology, First Affiliated Hospital of Kunming Medical University, Kunming, 650032 China; 3 College of Pharmaceutical Sciences, Yunnan University of Traditional Chinese Medicine, Kunming, 650500 China

**Keywords:** APS, Thrombosis, HIV

## Abstract

**Background:**

Antiphospholipid syndrome (APS) is a non-inflammatory autoimmune disorder induced by antiphospholipid antibodies, which occurs exceedingly rarely in pediatric population and even more rarely reported in HIV positive children.

**Case summary:**

A case of 11 years old boy had a sudden onset of swelling in his left lower leg along with pain which were worsening gradually. Initially, topical ointment was applied for 1 month which were ineffective in reducing pain and swelling. Instead, the symptoms were aggravated and suddenly spread to the proximal thigh, accompanied by dyskinesia of left lower leg. Both color doppler ultrasonography and vascular CT scan of left lower leg revealed deep venous thrombosis. His serum anti-phospholipid antibodies (aPLs) were tested positive. He was a known case of HIV virological failure with substantial HIV viral load (VL) despite receiving regular antiretroviral therapy (ART). His symptoms improved after giving aggressive antithrombotic and high dose corticosteroid treatments.

**Conclusion:**

When pediatric patients develop thrombotic disease, APS also needs to be ruled out. The autoantibodies levels should be routinely tested to look for recurrent thrombosis in children with HIV/AIDS.

## Background

Antiphospholipid syndrome (APS) which is also named as Hughes Syndrome, is an autoantibodies-induced autoimmune disease characterized by recurrent arterial or venous thrombosis and/or fetal loss associated with thrombocytopenia and persistently seropositive (usually moderate to high titer elevated) for anti-phospholipid antibodies (aPLs) [1]. The presence of circulating aPLs, including anticardiolipin antibodies (aCLs), lupus anticoagulant (LA) and/or anti-beta 2 glycoprotein I (β2GPI), are risk factor for developing thrombosis in asymptomatic individuals. It has been estimated that approximately 39% APS patients coexist with venous thromboembolism [2]. The existing studies have showed that HIV infected patients have 2–10-folds increased risk of developing thrombosis as compared with general population [3]. However, the physiological mechanism of the presence of coexisting APS and HIV infection affecting thrombosis process has not been well studied. Herein, we have reported a rare case of a coexistence of APS and asymptomatic HIV-infected child who has developed left lower extremity thrombosis.

## Case presentation

An 11 years old boy had a sudden onset of swelling in his left lower extremity along with pain which were worsening gradually for past 1 month duration. He had no history of fever, headache, cough, chest tightness, chest pain, abdominal pain and diarrhea. His CD4^+^ T lymphocyte cell count was 522 cells/μL and plasma VL was 943 copies/mL with a blood pressure around 97/69 mmHg at that time. Initially, topical ointment was applied for 1 month which were ineffective in reducing pain and swelling. Instead, the symptoms were aggravated and suddenly spread to the proximal thigh, accompanied by dyskinesia of left lower leg. The child was a known case of HIV positive since the age of two and was believed to be infected through mother to fetus transmission. He has been taking first-line regimen of ART (AZT + 3TC + NVP) since last 4 years when his CD4^+^ T lymphocyte cell count had reached 346 cells/μL at that time. The plasma HIV RNA was undetectable after half a year of ART.

His mother was HIV seropositive person. The child had no personal and/or family history of thrombotic diseases. There was no history of any prior surgery, trauma, prolonged bed rest, obesity, smoking and any other common risk factors of thromboembolic events.

On physical examination during hospital admission, the maximum circumference of his left thigh was 38 cm and the right thigh was 34 cm. The maximum circumference of his left calf was 27 cm and the right calf was 24 cm. His left lower thigh was inflamed and swollen, accompanied by tenderness and presence of varicose veins.

On hematological tests, his white blood cell (WBC) count was 3.28 × 10^9^/L with a CD4^+^ T lymphocyte cell count of 430 cells/μL, hemoglobin (Hb) was 126 g/L and platelets was decreased to 66 × 10^9^/L. On virology test, HIV RNA was found to be 580 copies/mL. The coagulation function test showed PT was reported to be 14.0 s and activated partial thromboplastin time (APTT) was prolonged to 44.3 s. The international normalized ratio (INR) and Fibrinogen (FIB) were 1.16 and 15.1 mg/L respectively. D-dimer was 6.26 mg/L. C-Reactive Protein (CRP) was 32.14 mg/L, procalcitonin (PCT) was 0.029 ng/ml and erythrocyte sedimentation rate (ESR) was 41 mm/h. The aCLs IgG was at 57 (normal < 22), IgM was at 24 (normal < 10). Anti-neutrophil cytoplasmic antibody (pANCA) and anti-β2GPI antibody were tested positive. In addition, some other autoimmune antibodies were also tested positive: ANA (1:100, granular); dsDNA: +; nucleosome: ++; histone: +; mitochondrial M2: +. Other examinations including liver and kidney function tests were unremarkable.

**Fig. 1:**
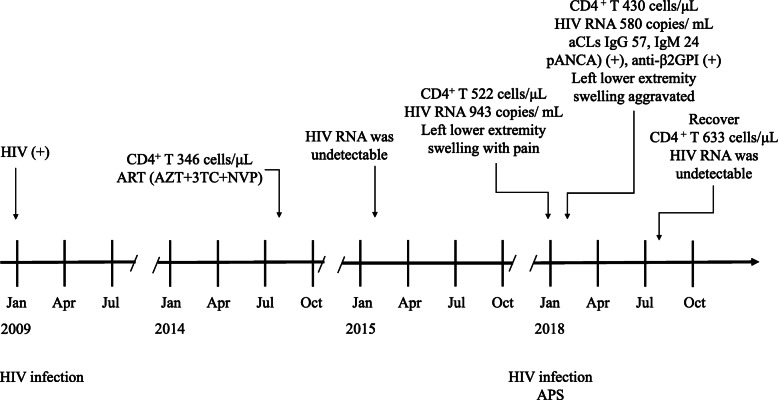
Timeline showing the clinical course of the patient with HIV infection and APS

Color doppler ultrasonography of lower extremity vessels revealed deep venous thrombosis in his left lower leg, accompanied by soft tissue edema and superficial lymphangiectasia. Vascular CT scan of left lower extremity showed extensive mural thrombosis (Fig. 1).

He was then diagnosed HIV complicated with thrombotic antiphospholipid syndrome. He was initially treated with low molecular weight heparin (LMWH) calcium 3000 IU every 12 h for 6 weeks followed by warfarin (1.25 mg/day) for long-term anticoagulant therapy at a target INR of 2.0–3.0. Besides, methylprednisolone (40 mg/day) treatment was also given to the patient for ten days which was substituted with 30 mg/day prednisone after that. The prednisone dosage was gradually reduced by 5 mg each time every 2 weeks until reached 5 mg/d and maintained for another 2 months. Additionally, antiviral regiment was adjusted to ABC + 3TC + LPV/r due to his persistent high HIV viremia.

**Fig. 2:**
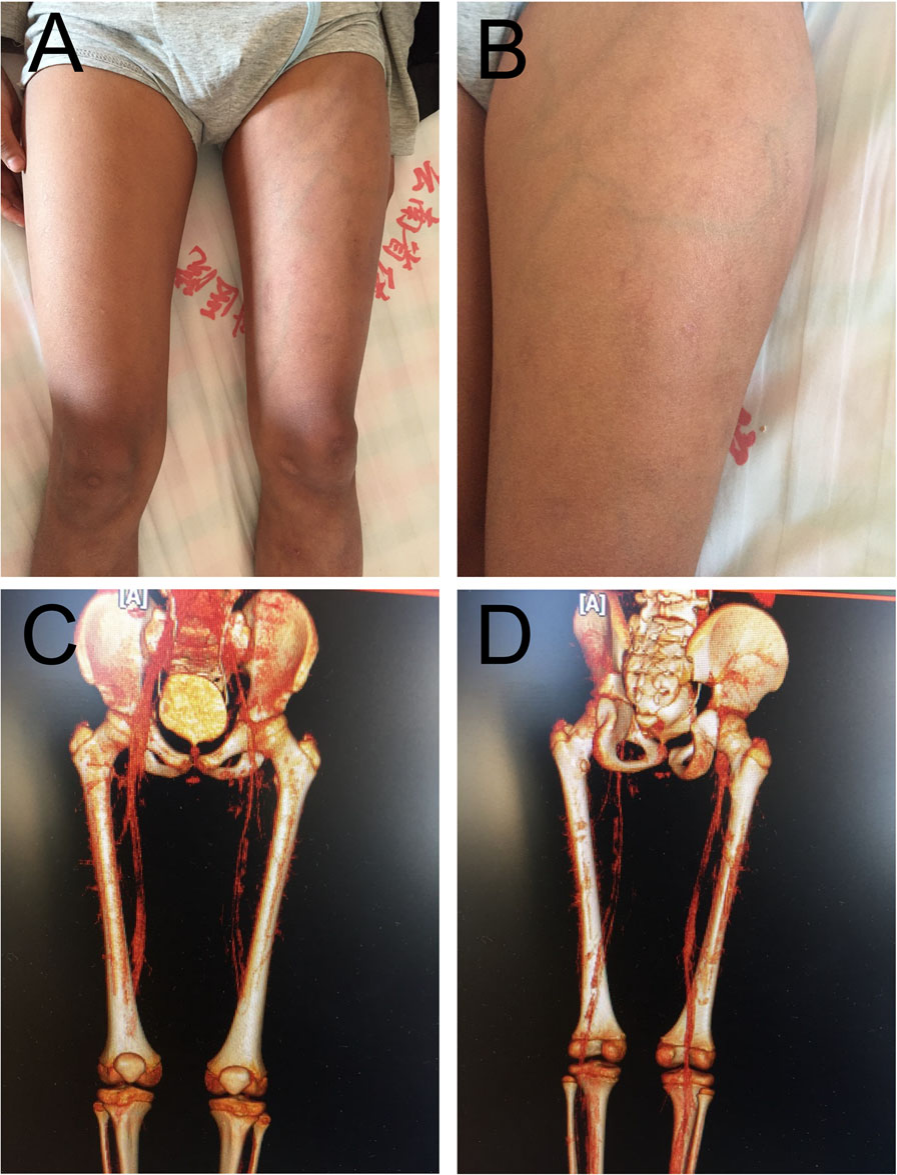
Clinical and Vascular CT three-dimensional reconstruction images of the patient. **a, b**. His left lower extremity thigh was inflamed and swollen with varicose veins. **c, d**. Vascular CT three-dimensional reconstruction showed left lower extremity showed extensive mural thrombosis

After one month of treatment, the swelling and pain were gradually relieved and vascular ultrasonography on follow up visit had showed the blocked vessel was partial recanalized and platelets count had returned to normal. After 3 months of treatment, his symptoms continued to be ameliorated. However, the aCLs and β2-GP1 antibody titers were still tested positive. After 6 months of treatment, his CD4^+^ T lymphocyte cell count was 633 cells/μL and HIV RNA was undetectable. His left lower extremity blood vessels were returned to normal. (Table 1 and Fig. 2).

**Table 1:** Clinical symptoms and Laboratory findings of the patient

Time	Clinical symptoms	Laboratory findings	Treatment regimens
January 2009	No	HIV (+)	–
August 2014	No	CD4^+^ T count: 346 cells/μL	ART (AZT + 3TC + NVP)
February 2015	No	HIV RNA: undetectable	ART (AZT + 3TC + NVP)
January 2018	Left lower extremity swelling with pain	CD4^+^ T count: 522 cells/μL, HIV RNA: 943 copies/mL	ART (AZT + 3TC + NVP)
February 2018	The maximum circumference of his left thigh was 38 cm and the right thigh was 34 cm. The maximum circumference of his left calf was 27 cm and the right calf was 24 cm. His left lower thigh was inflamed and swollen, accompanied by tenderness and presence of varicose veins.	CD4^+^ T count: 430 cells/μL, HIV RNA:580 copies/mL; **Ultrasonography and Vascular CT scan:** deep venous thrombosis in left lower extremity; **Coagulation function test:** PT: 14.0 s (normal, 11–16 s), APTT: 44.3 (normal, 23–40s), INR: 1.16, FIB: 15.1 mg/L (normal, 2-4 mg/L), D-dimer: 6.26 mg/L (normal, 0-1 mg/L); **Hematological tests:** WBC: 3.28 × 10^9^/L (normal, 3.5–9.5 × 10^9^/L), Hb: 126 g/L (normal, 130-175 g/L), PLT: 66 × 10^9^/L (normal, 125–350 × 10^9^/L; CRP: 32.14 mg/L (normal, 0-5 g/L), PCT: 0.029 ng/ml (normal, 0–0.046 ng/ml), ESR: 41 mm/h; **Autoimmune antibodies:** ACA (+), p-ANCA (+), β2-GP1-Ab (+), aCLs IgG: 57 (normal < 22), IgM: 24 (normal < 10)	ART (ABC + 3TC + LPV/r), LMWH calcium followed by warfarin; methylprednisolone followed by prednisone
August 2018	Left lower extremity revascularized	CD4^+^ T count: 633 cells/μL, HIV RNA: undetectable;	ART (ABC + 3TC + LPV/r)
		**Coagulation function test:** PT: 14.3 s (normal, 11–16 s), APTT: 21.4 (normal, 23–40s), INR: 1.19, FIB: 1.94 mg/L (normal, 2-4 mg/L), D-dimer: 1.12 mg/L (normal, 0-1 mg/L); **Autoimmune antibodies:** ACA (−), p-ANCA (−), β2-GP1-Ab (−), aCLs IgG: 19 (normal < 22), IgM: 4 (normal < 10)

## Discussion and conclusions

The etiology of APS is complex and has not been well delineated. The most popular hypothesis suggested a “two hit “ mechanism in pathogenesis of APS. The first hit is the presence of aPLs antibodies and interaction with vascular endothelial cells, neutrophils, platelets and monocytes resulting in endothelial dysfunction and injury. The second hit is the facilitation from predisposing factors like infections, contraceptives, malignancies and genetic susceptibility etc. which eventually leading to thrombotic events [4, 5]. Common infections associated with APS include typhoid bacillus, hepatitis virus, cytomegalovirus and particularly HIV infection [6–8]. Among APS patients, the incidence of HIV infection was as high as 17.0% [6].

The HIV virus, as a superantigen that may even resemble some autoantibodies, can induce APS through possible “molecular mimicry” mechanisms. Moreover, HIV virus can directly damage vascular endothelial cells and subsequent generation of autoimmune antibodies. Thus, participating the “double hit” pathway in the development and progress of APS [9, 10]. The patient we reported had experienced a virological failure with substantial HIV viral load (VL) despite receiving regular ART. It has been reported that the manifestation of APS was positively correlated with HIV viral load level [3, 11]. Some studies have even suggested HIV as a direct trigger of APS onset [12]. Therefore, the occurrence of APS may be associated with high levels of HIV viremia in HIV/AIDS patients.

The superimposed HIV infection might even further increase the risk of thrombosis in APS. The existing studies have showed that when compared with general population, HIV infected patients have 2–10-folds increased risk of developing thrombosis [8]. Firstly, HIV infection often leads to immunosuppression and affect B cell function resulting in increased production of autoantibodies [13]. Therefore, HIV-infected patients have higher concentrations of aPLs antibodies [14, 15]. Moreover, the deficiency of protein C and S and increased platelet activation are risk factors for thrombosis in HIV/AIDS patients [16–18]. Additionally, it has been reported that protease inhibitor (PI) in ART regimen might lead to thrombosis event as they could cause major lipid disturbances [19]. Therefore, the autoantibody levels should be routinely tested in HIV patients to alert for thromboembolic events.

APS is the principal cause of thrombotic disease in the pediatric population [20]. When thrombosis occurs in children, especially if it coexists with HIV infection, APS should be considered. While comprehensive reviewing the literatures, only three cases of HIV in children with APS were currently reported [21, 22]. Out of which, two cases couldn’t survive. Other patients that were reported thrombosis with APS and HIV were all adults [2, 7, 23–25]. Good result was achieved in our case through aggressive anticoagulant therapy and high-dose corticosteroids therapy.

HIV may induce the occurrence of APS and promote life threatening thrombotic events through several mechanisms. Therefore, the autoantibodies levels should be routinely tested in HIV/AIDS patients, especially in patients with virological failure with high levels of HIV viremia.

## Data Availability

Data sharing not applicable to this article as no datasets were generated or analyzed during the current study.

## Abbreviations

APS: Antiphospholipid Syndrome
VL: Viral Load
ART: Antiretroviral Therapy
aCLs: Anticardiolipin antibodies
LA: Lupus Anticoagulant
β2GPI: Anti-beta 2 glycoprotein I
WBC: White Blood Cell
Hb: Hemoglobin
APTT: Activated Partial Thromboplastin Time
INR: International Normalized Ratio
FIB: Fibrinogen
CRP: C-Reactive Protein
PCT: Procalcitonin
ESR: Erythrocyte Sedimentation Rate
pANCA: Anti-neutrophil cytoplasmic antibody
LMWH: Low Molecular Weight Heparin
